# Host defense alteration in *Caenorhabditis elegans* after evolution under ionizing radiation

**DOI:** 10.1186/s12862-024-02282-7

**Published:** 2024-07-09

**Authors:** Loïc Quevarec, Levi T. Morran, Elizabeth Dufourcq-Sekatcheff, Olivier Armant, Christelle Adam-Guillermin, Jean-Marc Bonzom, Denis Réale

**Affiliations:** 1 Institut de Radioprotection et de Sûreté Nucléaire (IRSN), PSE-ENV/SERPEN/LECO, Cadarache, Saint Paul Lez Durance, 13115 France; 2 Department of Biology, Emory University, Atlanta, GA 30322 USA; 3 Institut de Radioprotection et de Sûreté Nucléaire (IRSN), PSE-SANTE/SDOS/LMDN, Cadarache, Saint Paul Lez Durance, 13115 France; 4 Département des sciences biologiques, Université du Québec à Montréal, Montréal, QC Canada

**Keywords:** Experimental evolution, Evolutionary cost, Fitness, Host defense, *Caenorhabditis elegans*

## Abstract

**Background:**

Adaptation to a stressor can lead to costs on other traits. These costs play an unavoidable role on fitness and influence the evolutionary trajectory of a population. Host defense seems highly subject to these costs, possibly because its maintenance is energetically costly but essential to the survival. When assessing the ecological risk related to pollution, it is therefore relevant to consider these costs to evaluate the evolutionary consequences of stressors on populations. However, to the best of our knowledge, the effects of evolution in irradiate environment on host defense have never been studied. Using an experimental evolution approach, we analyzed fitness across 20 transfers (about 20 generations) in *Caenorhabditis elegans* populations exposed to 0, 1.4, and 50.0 mGy.h^− 1^ of ^137^Cs gamma radiation. Then, populations from transfer 17 were placed in the same environmental conditions without irradiation (i.e., common garden) for about 10 generations before being exposed to the bacterial parasite *Serratia marcescens* and their survival was estimated to study host defense. Finally, we studied the presence of an evolutionary trade-off between fitness of irradiated populations and host defense.

**Results:**

We found a lower fitness in both irradiated treatments compared to the control ones, but fitness increased over time in the 50.0 mGy.h^− 1^, suggesting a local adaptation of the populations. Then, the survival rate of *C. elegans* to *S. marcescens* was lower for common garden populations that had previously evolved under both irradiation treatments, indicating that evolution in gamma-irradiated environment had a cost on host defense of *C. elegans*. Furthermore, we showed a trade-off between standardized fitness at the end of the multigenerational experiment and survival of *C. elegans* to *S. marcescens* in the control treatment, but a positive correlation between the two traits for the two irradiated treatments. These results indicate that among irradiated populations, those most sensitive to ionizing radiation are also the most susceptible to the pathogen. On the other hand, other irradiated populations appear to have evolved cross-resistance to both stress factors.

**Conclusions:**

Our study shows that adaptation to an environmental stressor can be associated with an evolutionary cost when a new stressor appears, even several generations after the end of the first stressor. Among irradiated populations, we observed an evolution of resistance to ionizing radiation, which also appeared to provide an advantage against the pathogen. On the other hand, some of the irradiated populations seemed to accumulate sensitivities to stressors. This work provides a new argument to show the importance of considering evolutionary changes in ecotoxicology and for ecological risk assessment.

**Supplementary Information:**

The online version contains supplementary material available at 10.1186/s12862-024-02282-7.

## Background

Wildlife is currently confronted with multiple environmental stressors such as pollution or pathogens. Across generations, the populations can respond to these stressors by adapting through the selection of traits that are advantageous under these new conditions [1]. Potentially, this process allows populations to be adapted to local conditions, but poorly adapted to other conditions, i.e. adaptive cost [1–3]. Indeed, selection and adaptation come with a decrease in genetic diversity, which may decrease the chances of adapting to new stressors [4, 5]. Additionally, adaptation to new stressors may induce an evolutionary trade-off. This process describes situations where allocating more resources to a biological function reduces the resources provided to another function, or where genes involved in increasing the value of a fitness-related traits are also involved in reducing the value of another fitness-related trait [6–8]. Adaptive cost and evolutionary trade-offs can constrain the evolutionary trajectories of a population [8, 9]. For example, Dutilleul et al. [3] have shown that *Caenorhabditis elegans* populations evolving for 22 generations adapted to their salt or uranium environment. Also, populations evolving in a salt environment had lower fitness in a uranium environment, indicating an adaptive cost. In contrast, fitness in the salt environment was similar between uranium and salt exposed populations, indicating no adaptive cost for uranium-adapted populations. The identification and integration of these mechanisms are essential to characterize the long-term responses of populations to environmental stressors. However, the evolution and functioning of these trade-offs and associated costs is still relatively poorly understood empirically [8, 9].

The host defense, the protection of an organism against infections, is a trait relevant to study adaptive costs and trade-offs. Studies have observed evolutionary trade-offs between host defense and life history traits in bacteria (*Pseudomonas syringae* [10]), plant (*Arabidopsis thaliana* [11]) and animals (*Biomphalaria glabrata* [12], *Drosophila melanogaster* [13, 14], *Gallus domesticus* [15]). The presence of these trade-offs may be related to the fact that host defense is both costly and essential to the survival of the organism [16, 17]. Organisms continually interact with parasites that can impose detrimental effects to them. These interactions favor selection and evolution of high diversity of defense mechanisms to increased host defense to maintain their fitness under infection [18]. However, host defense is resource expensive with costs of upregulating the immune system upon parasitic infection (inducible costs) and costs of maintaining immunological machinery even lacking parasitism (constitutive costs) [19, 20]. However, the mechanisms inducing these costs associated with host defense are poorly understood. For example, several studies did not observe significant costs of host defense on life history traits when populations were exposed to parasites (*Caenorhabditis elegans* [18], *Drosophila melanogaster* [21, 22], *Poecilia reticulata* [23], *Trichoplusia ni* [24]). The absence of detection of host defense costs can be related to a lack of power related to experimental noise (e.g., lack of replicates, sample size too small…) or a lack of genetic variation on traits [18]. Alleles that confer greater defense could be strongly selected for in previous exposure and could lead to selective sweeps. In this case, the lack of diversity within the host population could prevent the observation of a trade-off [18]. Besides, environmental variables could influence the detection of defense costs in host-parasite system [25]. For example, Sandland and Minchella [25] have shown a trade-off between immune responses and life history traits in *Lymnaea elodes* population exposed to the parasite *Echinostoma revolutum*. The detection of this trade-off depended on nutrient availability. Studies investigating the costs associated with the host defense rarely incorporate other variations in environmental conditions [20], which may expose the existence of trade-offs.

The study of costs and trade-offs on the immune response in the presence of ionizing radiation is particularly relevant. Indeed, ionizing radiation and the oxidative stress it induces can have negative impacts on the host defense, even causing immunosuppression at the highest doses [26, 27]. These negative impacts are observed on innate and acquired immunity at molecular (gene expression, antibodies, antigens, cytokines…), cellular (leucocytes, T cells…) and tissue level (spleen, thymus, marrow…) [28–30]. On wildlife, rare studies in the radio-contaminated Chernobyl exclusion zone (Ukraine) have shown a decrease of immune response in *Hirundo rustica* populations and identified evidence of oxidative stress and immunosuppression in *Myodes glareolus* populations [31, 32]. Furthermore, a previous irradiation can alter host defense to parasites. Liu et al. [33] have shown, for example, a decrease of *C. elegans* survival to *Pseudomonas aeruginosa* after gamma irradiation at 50 Gy for one generation. In contrast, studies have shown that ionizing radiation can also stimulate host defense and immune response [34]. For example, Seong et al. [35] have shown an increased survival rate of *Drosophila melanogaster* to *Staphylococcus aureus* or *Pseudomonas aeruginosa* after being irradiated at 0.2 Gy with gamma rays for one generation. Similarly, Kimura et al. [36] observed an activation of innate immunity genes and an increased survival of *C. elegans* to *Pseudomonas aeruginosa* after being irradiated (X-rays) at 100 Gy for one generation. The difference between the results of Liu et al. [33] and Kimura et al. [36] might be explained by the type and method of irradiation (Liu et al. [33]: gamma irradiation, chronic exposure at 0.42 Gy.h^− 1^, 50 Gy total, as exposure to *Pseudomonas aeruginosa*; Kimura et al. [36]: X-rays, acute pre-treatment (x3) exposure at 789 Gy.h^− 1^, 100 Gy total, before exposure to *Pseudomonas aeruginosa*). Host defense plays an unavoidable role in survival and fitness, influencing evolutionary trajectory of populations. In a context of ecological risk assessment of radioactive pollution, it is therefore relevant to study the evolutionary responses of the host defense to this environmental stressor. However, to the best of our knowledge, the effects of evolution in gamma-irradiated environment, particularly adaptive cost on host defense have never been studied.

In previous studies, we showed that long-term irradiation can modify life history traits in *C. elegans* (e.g., sex ratio, population growth rate, hatching success or fecundity) and alter evolutionary trajectories through selection and adaptation mechanisms [37, 38]. In this study, we investigated (1) whether evolution in gamma-irradiated environment could induce a cost on survival against a second stressful environment, here with bacterial parasite *Serratia marcescens*. Furthermore, (2) we tested whether this succession of stressors induced the appearance of an evolutionary trade-off between fitness of irradiated populations and host defense. For this purpose, we chronically exposed *C. elegans* populations to 0, 1.4, and 50.0 mGy.h^− 1^ ionizing radiation throughout about 17 generation*s*. We then placed the populations in a common garden for about 10 generations. Next, we exposed the populations to the bacterial parasite *S. marcescens* for one generation and population survival was measured to estimate the effectiveness of their host defense according to the irradiated environment in which they had previously evolved. Finally, we tested for the presence of a negative correlation between fitness at the end of the multigenerational experiment and *C. elegans* survival to *S. marcescens*, suggesting the existence of an evolutionary trade-off.

Adaptation to a stressful environment can be associated with costs [3], notably on immunity, which is particularly costly in terms of resources [19, 20]. We hypothesized (1) that populations that have evolved in a pathogen-free irradiated environment, *a priori* without selective pressure on immunity, will show less effective defense against a pathogen challenge than control populations. (2) Furthermore, according to the trade-off hypothesis, independent of the treatment, populations with a higher relative fitness at the end of the multigenerational experiment (transfer 17) will have less effective defense and thus survive less to pathogen exposure.

## Results

We studied the effect of ionizing radiation on population fitness and its evolution over generations using a multigenerational experiment (part 1). Then, to assess whether the adaptive costs of evolution in an irradiated environment, we exposed the populations to a second stressor, the pathogen *S. marcescens*, and their defense was assessed through their survival rate (part 2). Finally, we verified the presence of an evolutionary trade-off between the level of adaptation of populations to ionizing radiation and resistance to the pathogen, i.e. do the populations that have invested the most in resistance to ionizing radiation have less resistance to the pathogen? To this end, we studied the correlation between the standardized fitness of each population at transfer 17 and the survival rate against the pathogen (part 3).

### Decreased fitness in response to ionizing radiation, but with improvement over time

**Fig. 1:**
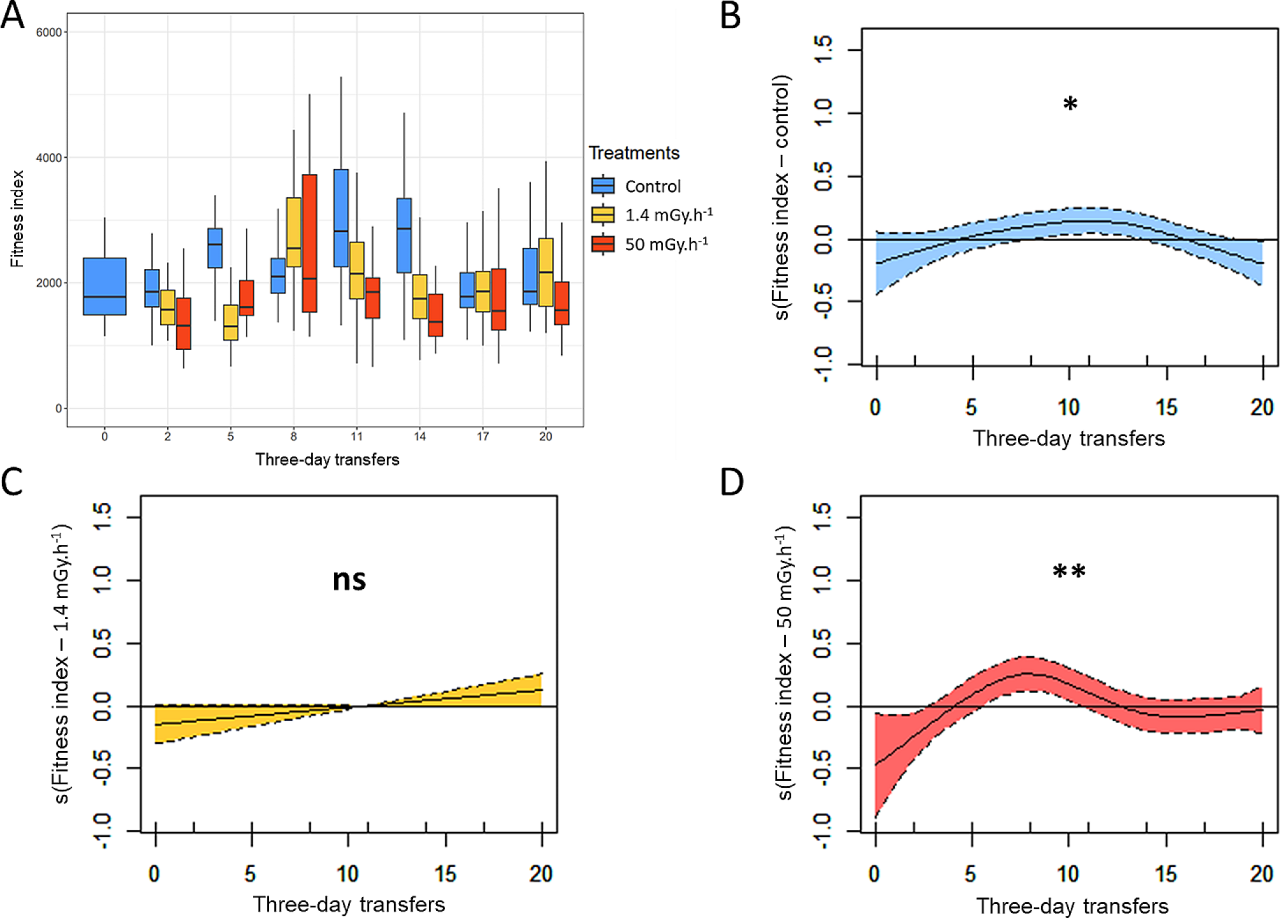
(**A**) Boxplot of fitness index over time (i.e., three-day transfers: 0, 2, 5, 8, 11, 14, 17 and 20) for *C. elegans* populations living in different gamma radiation environments. Blue: control; yellow: low radiation (1.4 mGy.h^− 1^); red: high radiation (50.0 mGy.h^− 1^). The data were analyzed using a GAMM and all results are presented in Table 1a. Briefly, fitness index was lower for 1.4 mGy.h^− 1^ (− 18%, p-value = 0.0007) and 50.0 mGy.h^− 1^ (− 28%, p-value = 2.36 e-08) compared with the control treatment (intercept in Table 1a). For transfer 0, *n* = 6; from transfers 2 to 20, *n* = 30 per treatment and per transfer. (**B**) to (**D**) GAMM graphical representation of the partial effects of generation on the fitness index for **(B**) Control (**C**) 1.4 mGy.h^− 1^ and (**D**) 50.0 mGy.h^− 1^. Shaded areas and dashed lines represent 95% confidence intervals. Partial response curves showing the relationship of the partial residuals of the response variable on the linear predictor scale and the relevant explanatory variables of the best approximate model. Plots were centered to have a mean value of zero along the y-axis, and the trends rather than the values of the plots were used to describe the responses to the smoothed explanatory variables. Variations observed for control (p-value = 0.017) and 50.0 mGy.h^− 1^ (p-value = 0.003) are significant. Results are present in Table 1b. ns: no significant; *, *P* < 0.05; **, *P* < 0.01; ***, *P* < 0.001

The fitness index (estimated as realized fecundity x survival rate) was estimated across all transfers at 2323, 1902 and 1667 for control, low and high irradiation treatments, respectively (Table 1a; estimations have been transformed with the inverse-log function). Globally, fitness index was significantly lower in the low (-18%, p-value = 0.0007) and high (-28%, p-value = 2.36 e-08) irradiation treatments than the control treatment (intercept) (Table 1a; Fig. 1). In the control populations, fitness index varied significantly across transfers (p-value = 0.017; Table 1b) increasing slightly from transfers 1 to 10 and decreasing slightly from transfers 10 to 20 (Fig. 1a and b). The fitness index was similar between the first and last transfers (Fig. 1b). Fitness index increased significantly until transfer 8, then decreased slightly up to transfer 11 then stabilized in high irradiation treatments (p-value = 0.003; Table 1b). The fitness index increased between the first and last transfers (Table 1b; Fig. 1a and d). In the low irradiation treatments, fitness index did not vary significantly across transfers (Table 1b; Fig. 1a and c).

**Table 1:** Effects of (**a**) gamma irradiation treatment (0.0, 1.4 and 50.0 mGy.h^− 1^) and (**b**) time (EDF: effective degrees of freedom) on *C. elegans* population fitness index during the 20 transfers of a multigenerational experiment. Ns: no significant; *, *P* < 0.05; **, *P* < 0.01; ***, *P* < 0.001

a)	Estimate	Std. Error	t value	Pr(>|t|)	
(Intercept)	7.750	0.042	185.35	< 2.00 e-16	***
Low radiation	-0.200	0.059	-3.39	0.0007	***
High radiation	-0.331	0.059	-5.66	2.36 e-08	***
Approximate significance of smooth terms					
**b)**	**edf**	**Ref.df**	**F**	**p-value**	
s(Time): Control	2.409	2.409	3.678	0.017	*
s(Time): Low radiation	1.000	1.000	3.689	0.055	ns
s(Time): High radiation	3.470	3.470	4.223	0.003	**

### Decreased host defense of previously irradiated populations

**Fig. 2:**
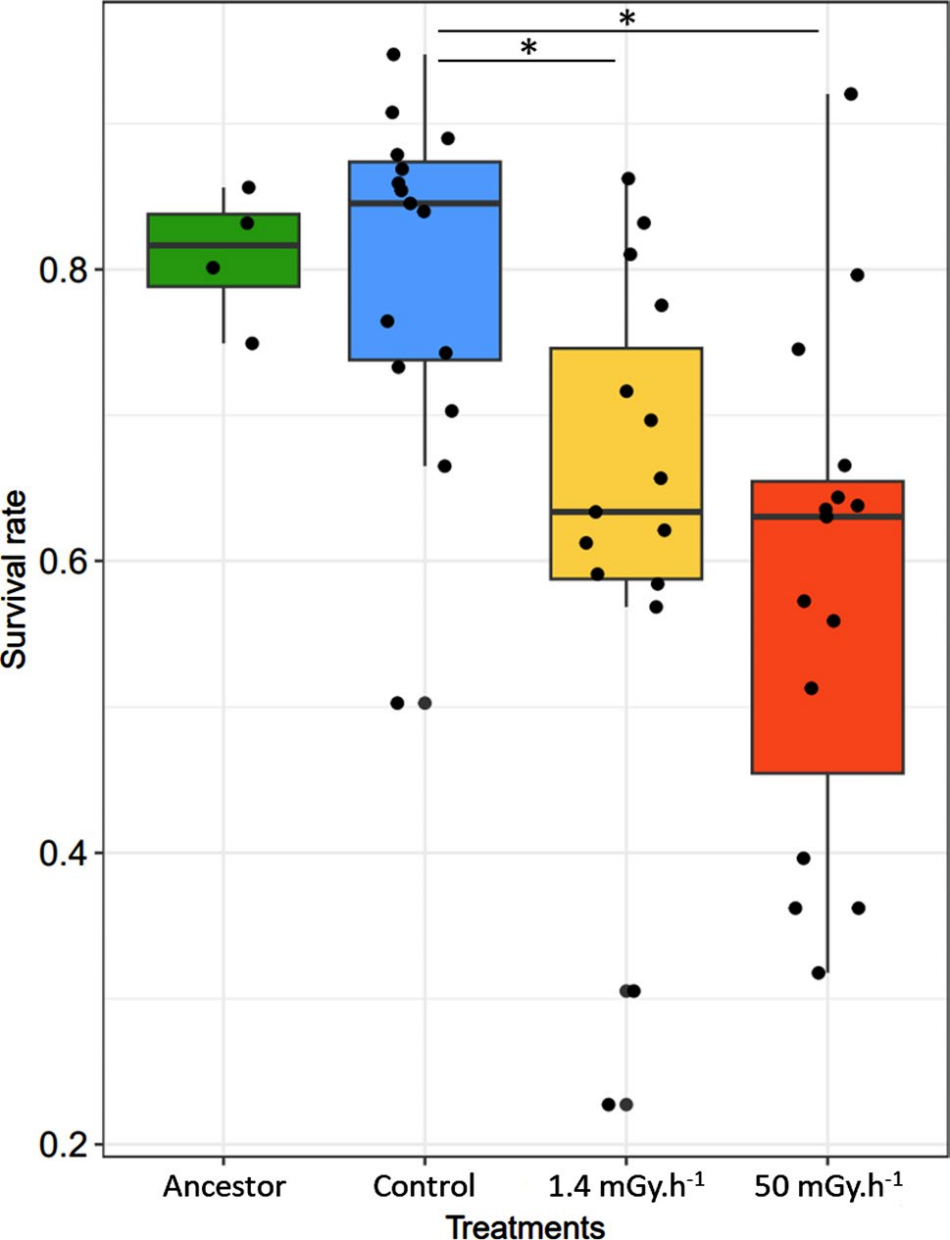
Boxplot of survival rate after two days of exposure to *S. marcescens* for *C. elegans* from the ancestral population (*n* = 4; green; at transfer 0) or from gamma irradiated populations at transfer 17 (Blue: control (*n* = 15); yellow, low radiation, 1.4 mGy.h^− 1^ (*n* = 15); red, high radiation, 50.0 mGy.h^− 1^ (*n* = 15)). The black dots correspond to the different measurements for each treatment. The data was analyzed using a GLMM; the results are present in Table 2. *, *P* < 0.05; **, *P* < 0.01; ***, *P* < 0.001

Survival rate was estimated at 0.81 for the ancestor population, 0.81 for the control, and 0.64 and 0.59 for the low and high irradiation treatments, respectively (Table 2). The irradiated populations showed a significantly lower proportion of live individuals than the control populations (Table 2). This corresponded to 17% decline in survival in the low (p-value = 0.042) and a 22% decline in survival in the high irradiation treatment (p-value = 0.013) compared to the control populations (Fig. 2). The ancestral population did not differ significantly from the control populations (Table 2; Fig. 2).

**Table 2:** Effect of original environment on survival rate after two days of exposure to *S. marcescens* for *C. elegans* populations from transfer 0 (ancestor) or from transfer 17 that evolved in different gamma radiation environments (0.0, 1.4 and 50.0 mGy.h^− 1^). Estimated variance of random effects = 3.476. Ns: no significant; *, *P* < 0.05; **, *P* < 0.01; ***, *P* < 0.001

	Value	Std. Error	DF	t-value	*p*-value	
(Intercept)	1.453	0.269	33	5.402	0.000	***
Low radiation	-0.857	0.376	12	-2.283	0.042	*
High radiation	-1.071	0.369	12	-2.903	0.013	*
Ancestor	-0.005	0.620	12	-0.008	0.994	ns

### Evolution towards cross-resistance to irradiation and pathogens

**Fig. 3:**
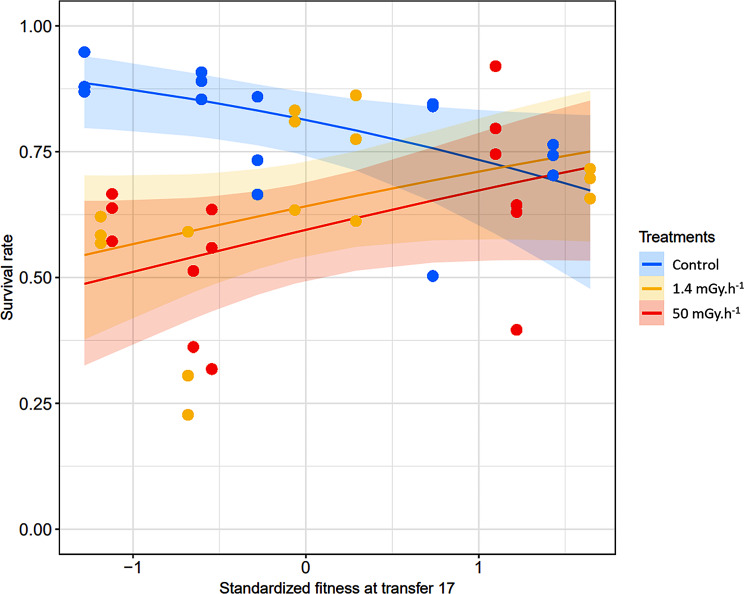
This graphical representation is estimated by GLMM. Lines showing the relationship between survival rate after two days of exposure to *S. marcescens* and standardized fitness for *C. elegans* populations at transfer 17 that evolved in different gamma radiation environments. Shaded areas represent 95% confidence intervals. The dots correspond to the values for the two traits for each population (5 populations with one fitness measurement for each x 3 survival measurements = 15 measurements per treatment). Blue: control; yellow: low radiation (1.4 mGy.h^− 1^); red: high radiation (50.0 mGy.h^− 1^). All the results of the statistical test are shown in Table 3. Briefly, we observed a negative correlation between survival rate and standardized fitness at transfer 17 for control treatment (p-value = 0.036), and a positive correlation for 1.4 mGy.h^− 1^ (p-value = 0.012) and 50.0 mGy.h-1 (p-value = 0.009)

The link between standardized fitness at transfer 17 and survival rate in response to infection differed according to the irradiation treatment. For the control treatment, survival of infected worms decreased significantly with standardized fitness at transfer 17 (p-value = 0.036; Table 3; Fig. 3), whereas it increased for both irradiation treatments (p-value = 0.012 and 0.009, respectively; Table 3; Fig. 3). Survival rate was estimated at 0.81 for the control, and 0.64 and 0.59 for the low and high irradiation treatments, respectively (Table 3).

**Table 3:** Effect of gamma irradiation treatment (0, 1.4 and 50 mGy.h^− 1^) and standardized fitness at transfer 17 on survival rate after two days of exposure to *S. marcescens* for *C. elegans* populations from transfer 17. Estimated variance of random effects = 0.212. *, *P* < 0.05; **, *P* < 0.01; ***, *P* < 0.001

	Estimate	Std. Error	z value	Pr(>|z|)	
(Intercept)	1.471	0.211	6.988	2.78e-12	***
Low radiation	-0.888	0.297	-2.990	0.003	**
High radiation	-1.087	0.297	-3.666	0.0002	***
Fitness	-0.456	0.218	-2.093	0.036	*
Low radiation: Fitness	0.771	0.308	2.506	0.012	*
High radiation: Fitness	0.794	0.307	2.591	0.009	**

## Discussion

We showed that ionizing radiation decreased the fitness of *C. elegans* populations (Table 1; Fig. 1). Fitness index was 18% and 28% lower on average for low and high irradiation treatments compared to the control treatment, respectively, but fitness index increased over time in the high irradiation treatments (Table 1b; Fig. 1d). When exposed to the bacterial parasite *S. marcescens* populations that have evolved in an irradiated environment showed a decline in survival by 17 and 22% for low and high irradiation treatments, respectively, compared to the control populations (Table 2; Fig. 2). These results validate our hypothesis that evolution in an irradiated environment has induced a cost by reducing the effectiveness of host defense. Next, we observed a negative correlation between standardized fitness at transfer 17 and host defense only for the control treatment, and a positive correlation for the two irradiated treatments (Table 3; Fig. 3). These results partly contradict our initial hypothesis of the presence of a trade-off between relative fitness at the end of the multigenerational experience (transfer 17) and survival to pathogen exposure, independently of treatment. We have shown that this trade-off was present without any stressor, and that in irradiated conditions we observe an evolution towards a cross-resistance to the two stressors, without, however, reaching a level of fitness and defense better than the populations of the control treatment.

### Decreased fitness in response to ionizing radiation, but with improvement over time

In the multigenerational experiment, our results showed a lower fitness index in two irradiated treatments compared to the control, and this effect increased with the dose rate. To our knowledge, studies describing the effects of ionizing radiation on evolution of fitness are scarce [39]. However, some studies have shown a decrease in reproduction following irradiation in many species [40, 41], specifically in *C. elegans* from 42.7 mGy.h^− 1^ [42–45], and in a multigenerational experiment from 1.4 mGy.h^− 1^ with the *C. elegans* A6140 population [38]. Also, studies have shown a decrease in survival following irradiation in *C. elegans*. Clejan et al. [46] showed a decrease of about 55% in the survival rate of *C. elegans* N2 strain exposed to an acute ionizing radiation dose of 60 Gy. We demonstrated a 7% decrease in larval survival in populations of *C. elegans* A6140 exposed for 20 generations to a dose rate of 50 mGy.h^− 1^ [38]. These results corroborate that a dose rate of 50 mGy.h^− 1^ has a negative impact on the population fitness of *C. elegans*. For 1.4 mGy.h^− 1^, the decrease in fitness in *C. elegans* is consistent with our previous studies, where we observed a decrease in realized fecundity for 20 generations to a dose rate of 1.4 mGy.h^− 1^ [38]. In contrast, no effect was observed in *C. elegans* N2 strain on hatching success between 6.6 and 45 mGy.h^− 1^ for shorter experiments (three generations [42]; one generation [47]), and on the number of larvae per hermaphrodite at 28 mGy.h^− 1^ [42]. However, other studies on gamma radiation have shown a decrease of survival of larvae at much lower chronic dose rates in other worms: 0.19 mGy.h^− 1^, 3.2 mGy.h^− 1^ and 4 mGy.h^− 1^ in *Neanthes arenaceodentata* (Polychaeta), *Ophryotrocha diadema* (Polychaeta) and *Eisenia fetida* (Oligochaeta), respectively [48–50].

After an initial decrease, our results also showed that fitness index increased until transfer 8 then stabilized in high irradiation treatments. Results and the shape of the curve suggested a local adaptation of populations to ionizing radiation, as described by Silander et al. [51]. Authors showed that fitness of populations adapting to a constant environment reaches a plateau due to a change in the ratio of beneficial and deleterious mutation rates. Transgenerational effects could also explain fitness changes in high irradiation treatments. For example, Yue et al. [52] has shown oscillatory changes in *C. elegans* reproduction exposed for 11 generations to 1-ethyl-3-methylimidazolium bromide-related transgenerational effect. For low irradiation treatments, the similar but nonsignificant increase observed for fitness index suggest process in the same direction but slowed at the low dose rate. These results contrast with our previous studies, where we observed an adaptive response on embryo survival and a slower life history of populations that live in low irradiation treatments [38]. According to the intensity of ionizing radiation, these opposing conclusions suggested that evolutionary response (i.e., transgenerational effects or adaptation) could take place on different traits. Indeed, the response of organisms may differ according to the ionizing radiation dose [53–55].

Finally, results showed a slight increase in fitness index up to transfer 10 for the control, in the same way as for the irradiated treatments. However, in contrast to treatment 50 mGy.h^− 1^, where an increase in fitness index was observed between the first and last transfers, for the control treatment no increase was observed. These results showed that part of the observed variation was probably due to environmental noise, but that this explanation was not sufficient to explain the improved fitness in the irradiated treatment, suggesting an evolutionary response in highly irradiated populations.

### Decreased host defense of previously irradiated populations

The defense of the organism is essential for its survival and can be estimated by exposing individuals to a pathogen [56]. We showed that *C. elegans* populations that evolved in an irradiated environment survived less well to *S. marcescens* than control populations (Fig. 2). We observed a decrease in survival rates for common garden populations that had previously evolved under both irradiated treatments. These results suggested that long-term exposure to ionizing radiation decreased the effectiveness of host defense. These changes may have a genetic cause, since populations from the different irradiation treatments (0.0, 1.4 and 50.0 mGy.h^− 1^) were placed in common garden conditions for 10 generations before the beginning of exposure to the pathogen. Indeed, *C. elegans* shows large variation in its responses to pathogenic bacteria, notably linked to genetic variability [18, 57, 58]. However, we cannot exclude that at least part of the changes was related to long-term transgenerational epigenetic effects [59]. This suggests that adaptation to ionizing radiation was associated with an evolutionary cost on the effectiveness of host defense. Several studies have also shown deleterious effects of ionizing radiation on defense of the organism in one-generation experiment. Liu et al. [33] found a decrease in survival of *C. elegans* infected with *Pseudomonas aeruginosa* when irradiated at a dose of 50 Gy with gamma radiation. Rossmoore and Hoffman [60] showed increased mortality of *O. leucostigma* larvae exposed to the pathogen *Bacillus thuringiensis* ten days after a gamma irradiation acute dose of 300 Gy. More specifically, studies showed deleterious effects of ionizing radiation on immune response in *Oncorhynchus mykiss* at 4.66 mGy.h^− 1^ on humoral immune response [61], in *Myodes glareolus* between 15 µGy.h^− 1^ and 18 µGy.h^− 1^ on pathways associated with immunity (e.g., impaired antigen processing and activation of leucocytes involved in inflammatory responses) [32] or in *Hirundo rustica* between 2.6 µGy et 4.4 µGy on lymphocytes, immunoglobulin and spleen [31]. Interestingly, control populations had a similar survival rates than the ancestral population, suggesting that without the stressor, the immune response remained stable over time.

### Evolution towards cross-resistance to irradiation and pathogens

Host defense and reproduction of the organism require a significant resource investment [62], and that frequently a trade-off in resource allocation exists between these two functions (see introduction). Thus, it is particularly relevant to study whether an evolutionary trade-off exists between fitness and the immune response. Only control populations showed a negative relationship between standardized fitness at transfer 17 and survival following bacterial infection. In contrast the low and high irradiated populations with the highest relative fitness at transfer 17 also showed the highest survival when infected (Fig. 3; Table 3). These results indicate the presence of an evolutionary trade-off between fitness in presence and in absence of *S. marcescens* pathogen, for the populations in the control treatment. Investment in life-history traits that improve reproduction and offspring survival is associated with a reduction in defense efficiency in *C. elegans*. This result was consistent with numerous studies demonstrating negative relationship between reproduction and immunity efficiency in birds [63], insects [17], gastropods [25] or the nematode *C. elegans* [64, 65]. Furthermore, studies have demonstrated in *C. elegans* and *D. melanogaster* that the absence of reproduction linked to the lack of a germline increases resistance to various pathogens [66, 67]. Reproduction and immune responses are both energetically costly, the existence of a trade-off between the two traits is probably linked to an alternative allocation of limiting energetic resources [17].

For both irradiated treatments, we did not observe any evolutionary trade-off; on the contrary, the *C. elegans* populations with the best fitness in irradiated environments also survived better against *S. marcescens*. This result seems to indicate that during the multigenerational experiment, selection for traits related to improve resistance to ionizing radiation may also lead to improved host defense, i.e. exaptation [68]. Similarly, research has shown that populations of *C. elegans* evolved increased resistance to uranium and NaCl salt after evolving for 22 generations in an environment containing uranium [3]. In the case of heavy metals, several authors [3, 69] explain that this cross-resistance can be explained by detoxification mechanisms common to both stressors. Besides, these authors suggest that cross-resistance may be caused by a single major gene or a few genes with effects specific to a class of pollutants or even to more general pollutant actions. In our case, we observe a cross-resistance between gamma irradiation and a pathogen, which has already been described in *C. elegans* [70, 71]. Ermolaeva et al. (2013) have shown that DNA damage induced by ionizing radiation or UV irradiation triggers a cascade that activates innate immunity, for example by increasing the expression of antimicrobial peptides. Thus, if evolutionary changes were to alter this cascade, we might expect changes in both resistance to irradiation and to defense against pathogens. In parallel, this positive relationship for both irradiation treatments could reflect the effect of the accumulation of deleterious mutations differing between populations. Those with the lowest fitness would have more deleterious mutations or with greater effects. These same populations would also survive less well against *S. marcescens*. Thus, the accumulation of deleterious mutations induced by ionizing radiation would lead to increased long-term sensitivity of populations to stress factors, particularly pathogens.

While we showed that adaptation to ionizing radiation increased with survival to *S. marcescens* for irradiated populations, it is interesting to note a global decrease of fitness over the multigenerational experiment and in survival to *S. marcescens* for part of the irradiated populations compared with control populations, and thus an opposite response depending on the scale of the analysis, at least in appearance. These results could be explained by evolutionary mechanisms that produces random changes, specifically by an accumulation of deleterious mutations induced by radiation, known to be strongly mutagenic [72]. Moreover, the mutations that appear are much more likely to be deleterious than beneficial [73]. The presence of genetic drift could accentuate the fixation of deleterious mutations, resulting in a deterioration of the traits studied and a divergence of phenotypic responses between independent populations after several generations [74, 75]. This process could explain the greater variance in survival measurements to the pathogen for the 50 mGy.h^− 1^ treatment than for the control treatment (Fig. 2). Indeed, within the treatments, we observed different responses between the populations. For example, some had a survival rate to the pathogen comparable to the control populations, while others were much weaker (Fig. 3). This result could indicate that, in parallel with the accumulation of deleterious mutations, favorable mutations induced by irradiation could be selected in some populations in the two irradiated treatments.

## Conclusion

Our results showed that the fitness of irradiated *C. elegans* populations increased over time, but remains lower than fitness of control populations. The evolution in gamma-irradiated environment has resulted in greater susceptibility to the pathogen *S. marcescens*. This effect was amplified by increasing the dose rate. These results suggest that adaptation to ionizing radiation is associated with an evolutionary cost on the effectiveness of *C. elegans* defense. Furthermore, we showed a trade-off between standardized fitness at the end of the multigenerational experiment and survival of *C. elegans* to *S. marcescens* in the control treatment, and on the contrary a positive correlation between the two traits for the two irradiated treatments, indicating an evolution towards cross-resistance to the two stressors. In some populations, the evolution of resistance to ionizing radiation also seems to have been an advantage in defense against the pathogen. Despite the improvement in fitness and defense of *C. elegans* to *S. marcescens* in irradiated condition for some populations, globally the two traits remain weaker than the populations of the control treatment. Also, the most sensitive populations to long-term ionizing radiation have also become more susceptible to the pathogen. Organisms are often exposed to different stressors in time and space. Understanding these stressors on the evolutionary trajectories of populations and the associated costs is an important challenge. In an ecological risk assessment process, it is therefore important to ask whether a population can adapt to one or more stressors, and at what cost? Our study emphasizes this last point, since we have shown that even if a population seems to adapt to a stressor (here ionizing radiation), and that this also favors host defense, the evolutionary cost remains much higher overall. However, it also shows that if populations are given enough time to adapt, they seem to be able to recover a fitness level comparable to that of the unexposed populations.

## Methods

### Test organism and population maintenance

**Fig. 4:**
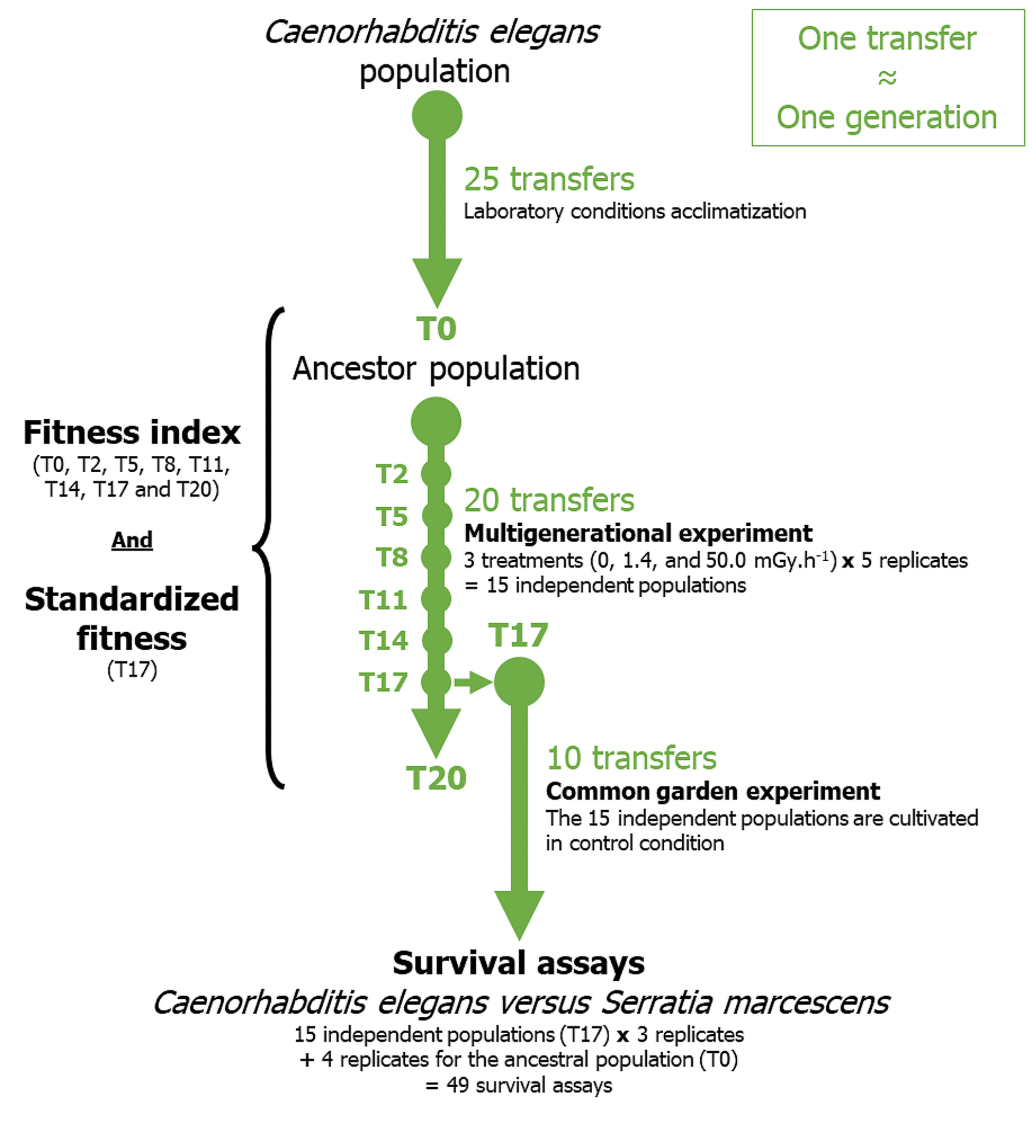
Schematic overview of the experiments protocol for *Caenorhabditis elegans* population A6140 under different gamma radiation treatments and the survival assays to *Serratia marcescens* (strain SM2170). After 25 three-day transfers (around 25 generations) to acclimate *C. elegans* population to laboratory conditions, fitness index was estimated at transfer 0, 2, 5, 8, 11, 14, 17 and 20 (multigenerational experiment) for populations exposed to three dose rate gamma radiation treatments: control (0.0 mGy.h^− 1^), low irradiation (1.4 mGy.h^− 1^), and high irradiation (50.0 mGy.h^− 1^). For each treatment, we created five independent populations replicates. We also estimated standardized fitness index (i.e., fitness for each population independently of treatment) at transfer 17 (T17). The 15 independent populations from transfer 17 and the populations from transfer 0 (ancestor – T0) were cultivated under control condition (common garden experiment) for 30 days (10 transfers), then were exposed to the bacterial parasite *Serratia marcescens* and their survival was estimated (survival assays) to study host defense

We cultured population of the androdioecious nematode *Caenorhabditis elegans* A6140 on 6 cm Petri dishes at 20 °C and 80% relative humidity to have a generation time of approximately 3 days [76]. The *C. elegans* population A6140 was created from a mixture of 16 wild isolates and is characterised by high genetic diversity and a male frequency of about 20% [77, 78]. We filled Petri dishes with 12 mL of nematode growth medium (NGM) and seeded with *Escherichia coli* bacteria (OP50 strain) ad libitum [37]. Before exposure to ionizing radiation, the stock population was maintained for at least 25 three-day transfers (around 25 generations) to acclimate to laboratory conditions (Fig. 4). Every three days, we washed nematodes off the Petri dishes with an M9 solution. We then estimated the number of individuals in six sample drops of 5 µL with a stereomicroscope (Olympus SZX12, 1.6 × 90 magnification) and we transferred 1000 nematodes at all developmental stages into two new dishes to ensure they were fed *ad libitum* (see [38]).

### Irradiation conditions

The external gamma radiation exposure was conducted at the Mini Irradiator for Radio Ecology (MIRE) ^137^Cs irradiation facilities, at the French Institute for Radioprotection and Nuclear Safety (IRSN, Cadarache, France). We used the same irradiation facilities and the same protocol as previously described by Quevarec et al. [37]. We placed the Petri dishes vertically in the irradiator to homogenize the dose received over the entire dish. Placing the plates at different distances from the source and separated by shields (Petri dish filled with lead filings) allowed us to obtain the required dose rates. The dose rates were measured with radiophoto luminescent (RPL) micro-dosimeters twice during the experiment. For control treatment, we placed the Petri dishes in an identical incubator, but without irradiation system.

### Multigenerational experiment: fitness index estimate

We used three dose rate gamma radiation treatments: control (0.0 mGy.h^− 1^), low irradiation (1.4 mGy.h^− 1^), and high irradiation (50.0 mGy.h^− 1^). Both irradiation treatment had an environmental relevance, for example in the Chernobyl exclusion zone (CEZ), Garnier-Laplace et al. [79] reported that terrestrial wildlife could be exposed to dose rates up to ∼10 mGy.h^− 1^ and Geras’kin et al. [80] estimated a dose rates up to ~ 110 mGy.h^− 1^ in the month following the accident, in the most contaminated 600 ha of the CEZ. For each treatment, we created five independent replicates taken from the stock population and maintained for over 60 days, with a transfer to new Petri dishes once every three days (Fig. 4). At the beginning of each transfer, each replicate contained initially 1000 worms equally distributed into two Petri dishes (initial density of 500 worms/plate).

For each treatment and transfer, we estimated fitness index (realized fecundity x survival rate) from 30 measures, corresponding to six measures (measurement group) for each biological replicate (five independent populations per treatment) (Fig. 4). Fitness index was estimated at transfer 0, 2, 5, 8, 11, 14, 17 and 20. Realized fecundity corresponded to the number of eggs / 1000 hatched individuals / unit time (i.e., from larval to adult stage; population estimated after 3 days of growth), based on the definition of Tarsi and Tuff [81]. Realized fecundity was estimated in six sample drops of 5 µL per replicate. Survival rate corresponded to the hatching success at transfer 0, 2, 5, 8, 11, 14, 17 and 20. For estimated hatching success, we transferred 100 eggs per replicate from washed Petri dishes that had contained the populations into a new 3 cm Petri dish with NGM. At the end of each three-day transfer, we washed the Petri dishes with M9 solution to collect and re-seed the populations on new Petri dishes (5 populations per treatment; as described previously). On the washed Petri dishes, some eggs remained attached to the NGM. We collected a hundred of these eggs per population and isolated them to quantify hatching success. As the populations were in the mix stage, the eggs could be freshly laid or no more than 10–12 h old at 20 °C [76]. Forty-eight hours after the transfer, we counted hatched nematodes (between 48 and 60 h after egg-laying, corresponding globally to the L4 and young adult stages) and estimated hatching success as the ratio of the number of hatched individuals on the number of eggs initially put on the Petri dish for each replicate. All measurements for realized fecundity and survival rate were performed with a stereomicroscope (see [38]).

### Estimation of the immune response — survival assays

At transfer 17, populations from each replicate and each treatment were cultured in control condition for about 10 generations (common garden experiment) and were sent to Morran’s laboratory at Emory University (Atlanta, USA) to study host defense (Fig. 4).

To estimate the effects of irradiation on *C. elegans* host immune response, we assessed the 48-hour survival of the experimental populations and of the ancestral population. We used the bacterial parasite, *Serratia marcescens* strain SM2170, to infect *C. elegans* on Serratia Selection Plates (SSPs) [82]. This parasite can induce a high mortality in *C. elegans* after colonizing the nematode’s intestine [83]. SSPs are 10 cm Petri dishes containing NGM-Lite agar (US Biological, Swampscott, MA). One side of the SSP was seeded with 30 µL of an overnight culture of SM2170. The opposite side of the SSP was seeded with 50 µL of an overnight culture of OP50 *Escherichia coli* to serve as a relatively benign food source for nematodes surviving parasite exposure. The plates were incubated at 28 °C for 24 h. Then, 20 µL of ampicillin (200 µg/mL) was applied to the middle of the plate in an area without SM2170 or OP50, for stopping the spread of SM2170 into OP50. Finally, we transferred approximately 200 (ranging from 154 to 336) L4 and young adult nematodes onto the SM2170 side of the plate and maintained on the SSP for 48 h at 20 °C. After 48 h, we counted the number of live worms on each SSP and determined the 48-hour survival rate for each technical replicate [56]. We measured survival rate as the number of living worms divided by the total number of worms plated.

We assayed 49 SSPs (4 SSPs replicates for the ancestral population (T0) + 3 SSPs replicates x 15 replicates of experimental populations (T17)), corresponding to 15 SSPs per treatment (0, 1.4, and 50.0 mGy.h^− 1^) and 4 for ancestral population.

### Evolutionary trade-off

At transfer 17, we estimated the standardized fitness of each population from the fitness index described above. We calculated standardized fitness *x* as:

$$x=\frac{X-\mu }{\sigma }$$

Where *X* is the value of fitness index to standardize, *µ* is the mean fitness index of the treatment (control, low or high irradiation) and *σ* is the standard deviation of fitness index to a treatment. This calculation allows us to compare the fitness of populations independently of the variation induced by the treatment. We studied the relationship between standardized fitness at transfer 17 of irradiated populations and survival in bacteria-infected populations, measured after the common garden experiment (Fig. 4).

### Statistical analysis

Before the analysis, we log-transformed data of fitness index (data in supplementary information; Table S1). We used a Mixed Generalized Additive Model (GAMM) with R software [84] and the Mgcv package [85] to analyze fitness index with Gaussian distribution. No overdispersion of the data was observed. We analyzed fitness index as a function of transfer (a continuous variable), irradiation treatment (control, low and high irradiation) and their interaction as fixed effects. ID of the replicate and measurement group ID were added as random effects. The smoothing was performed on the variable transfer in function of treatment.

We analyzed survival rate and the correlation between the survival rate of infected worms and the standardized fitness at transfer 17 with quasi-binomial and binomial (logit link function) distribution, respectively, using a Generalized Linear Mixed Model (GLMM) (lme4 [86] and MASS packages [87]). No overdispersion of the data was observed. Survival rate was analyzed as a function of irradiation treatment (ancestor, control, low and high irradiation) as fixed effects, and the ID of the replicate as a random effect (data in supplementary information; Table S2). For correlation between the survival rate and the standardized fitness, survival rate was analyzed as a function of irradiation treatment (control, low and high irradiation), standardized fitness, and their interaction as fixed effects and replicate ID as a random effect (data in supplementary information; Table S2).

Because we used GLMMs with logit link functions, we provide the estimated parameters in the text after back transforming the coefficient using the inverse logit function (untransformed coefficients are shown in the Tables). The log-transformed raw data of fitness index are also back transformed in the text.

### Electronic supplementary material

Below is the link to the electronic supplementary material.

Supplementary Material 1

## Data Availability

Data is provided within the manuscript or supplementary information files.
